# Bulk Rashba‐Type Spin Splitting in Non‐Centrosymmetric Artificial Superlattices

**DOI:** 10.1002/advs.202206800

**Published:** 2023-02-19

**Authors:** Woo Seung Ham, Thi Huynh Ho, Yoichi Shiota, Tatsuya Iino, Fuyuki Ando, Tetsuya Ikebuchi, Yoshinori Kotani, Tetsuya Nakamura, Daisuke Kan, Yuichi Shimakawa, Takahiro Moriyma, Eunji Im, Nyun‐Jong Lee, Kyoung‐Whan Kim, Soon Cheol Hong, Sonny H. Rhim, Teruo Ono, Sanghoon Kim

**Affiliations:** ^1^ Institute for Chemical Research Kyoto University Uji Kyoto 611‐0011 Japan; ^2^ Department of Physics University of Ulsan Ulsan 44610 Korea; ^3^ Japan Synchrotron Radiation Research Institute (JASRI) Sayo Hyogo 679‐5198 Japan; ^4^ International Center for Synchrotron Radiation Innovation Smart Tohoku University Sendai 980‐8572 Japan; ^5^ Center for Spintronics Korea Institute of Science and Technology (KIST) Seoul 02792 Korea

**Keywords:** artificial superlattice, bulk Rashba‐type spin splitting, charge‐to‐spin conversion, spin current

## Abstract

Spin current, converted from charge current via spin Hall or Rashba effects, can transfer its angular momentum to local moments in a ferromagnetic layer. In this regard, the high charge‐to‐spin conversion efficiency is required for magnetization manipulation for developing future memory or logic devices including magnetic random‐access memory. Here, the bulk Rashba‐type charge‐to‐spin conversion is demonstrated in an artificial superlattice without centrosymmetry. The charge‐to‐spin conversion in [Pt/Co/W] superlattice with sub‐nm scale thickness shows strong W thickness dependence. When the W thickness becomes 0.6 nm, the observed field‐like torque efficiency is about 0.6, which is an order larger than other metallic heterostructures. First‐principles calculation suggests that such large field‐like torque arises from bulk‐type Rashba effect due to the vertically broken inversion symmetry inherent from W layers. The result implies that the spin splitting in a band of such an ABC‐type artificial SL can be an additional degree of freedom for the large charge‐to‐spin conversion.

## Graphical abstract

Bulk Rashba‐type charge‐to‐spin conversion  occurs in a non‐centrosymmetric [Pt/Co/W] superlattice showing considerable W thickness dependency. Findings suggest that spin splitting in the non‐centrosymmetric artificial superlattice may be an additional degree of freedom for sizeable charge‐to‐spin conversion.

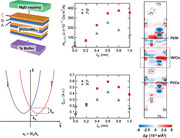

## 1 Introduction

Increasing demands for information processing have expedited the development of faster and more energy‐efficient magnetic data storage systems with a high capacity.^[^ ^1^ ^]^ Magnetization switching has been intensively studied for energy‐efficient writing schemes.^[^ ^2^, ^3^ ^]^ In this scheme, altering orthogonal magnetizations is directly related to the energy consumption in storing information. During the last decade, magnetization switching by the angular momentum transfer from spin current, known as spin‐orbit torque (SOT), has been intensively studied.^[^ ^4^, ^5^, ^6^, ^7^, ^8^, ^9^, ^10^, ^11^, ^12^, ^13^, ^14^ ^]^ Here, charge current should be converted to the spin current via spin Hall or Rashba effects.^[^ ^3^, ^4^, ^5^, ^15^, ^16^, ^17^, ^18^ ^]^ Therefore, a system with a large charge‐to‐spin conversion ratio (*ξ*_CS_) would be promising for spin‐based electronic devices with high energy efficiency. Although many heavy metals such as Ta, and W instead of Pt have been reported as candidates with sizable *ξ*_CS_, their high resistivity increases power consumption. Hence, a quest for materials or material combinations possessing larger *ξ*_CS_ with high conductivity is still required.^[^ ^5^, ^19^, ^20^ ^]^

**Figure 1:**
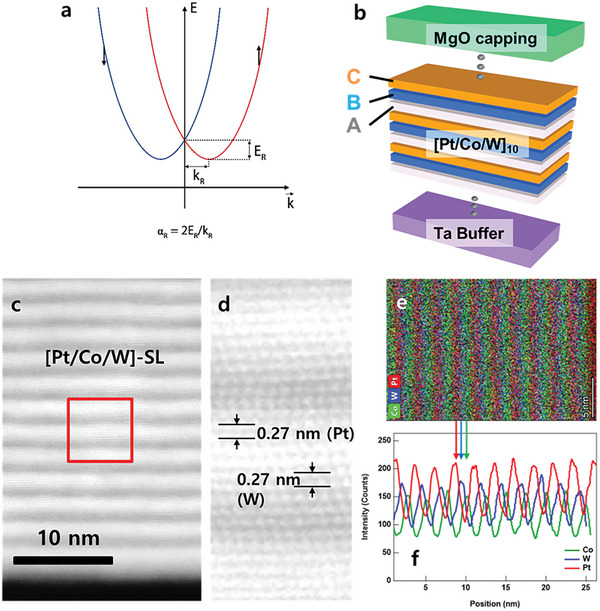
Rashba‐split band in an ABC‐type SL. a) Schematic image of the Rashba‐split band. b) Artificial SL with non‐centrosymmetric layer structure based on Pt, Co, and W. c) HAADF‐STEM image of the [Pt/Co/W (0.6 nm)] superlattice and d) enlarged image of the red‐squared area in (c). e) The energy‐dispersive spectroscopy (EDS) mapping image of the superlattice. f) the chemical profiles of Co, Pt, and W obtained from the EDS mapping. The arrows link the mapping image and the profiles with each element's information.

Heterostructures with inversion asymmetry at an interface are another pathway to generate the SOT as well as materials with large SOC. The inversion asymmetry in a heterostructure inherently creates the interfacial electric field, generating Rashba spin splitting in a momentum space. The Rashba effect is conveniently expressed by a Hamiltonian, $H_{R}=\alpha_{R}\left(k\times \sigma \right)\cdot \hat{z}$, which gives rise to the coupling between the electron spin *σ* and the crystal momentum *k*.^[^ ^21^ ^]^ Here, *α*_R_ is the Rashba parameter measuring the magnitude of the splitting or the strength of SOC; $\hat{z}$ is the unit vector along orthogonal to an interface. **Figure** **1**a schematically illustrates the band splitting due to the Rashba effect. The Rashba SOC interaction breaks the spin degeneracy, resulting in the formation of distinct red and blue parabolic bands shifted along the momentum axis. The Rashba parameter *α*_R_ is estimated as *α*_R_ = 2*E*_R_/*k*_R_, where *E*_R_ and *k*_R_ are Rashba energy and momentum offset, respectively. Recent reports have demonstrated that non‐centrosymmetric crystals, such as BiTeX (X = Cl, Br, and I), exhibit giant Rashba‐type spin splitting.^[^ ^22^, ^23^ ^]^ Also, bulk Rashba‐type spin splitting in non‐centrosymmetric‐artificial SLs has been reported to result in large perpendicular magnetic anisotropy (PMA) and Dyaloshinskii‐Moriya interaction (DMI).^[^ ^24^, ^25^ ^]^ However, finding materials with large Rashba effect in bulk is quite limited in practice. Hence, the fabrication of artificial SL with large Rashba effect by conventional vacuum technology offers a great advantage to realize the next‐level spin‐orbitronic devices.

Here, we demonstrate charge‐to‐spin conversion via Rashba‐type effect in a ferromagnet system, which is non‐centrosymmetric with an atomically layered structure. In this SL, two different crystal elements Pt and W composed of a few atomic monolayers are in contact with 6 Å Co layers, which gives rise to structures with broken inversion symmetry as illustrated in Figure 1b. This study shows that *ξ*_CS_ in the [Pt/Co/W]‐SL obtained from field‐like SOT, dominantly originating from Rashba‐Edelstein effect, is ≈0.59. This is 2–10 times larger than those of popular metallic bilayers such as Pd/Co, Pt/Co, Ta/CoFeB, and W/CoFeB^[^ ^5^, ^10^, ^11^, ^12^, ^13^ ^]^ as well as that of the [Pt/Co]‐SL. The [Pt/Co/W]‐SL even shows a good spin Hall conductivity of 7.0 × 10^5^±500 (ℏ/2*e*) (Ω m)^−1^ because of both *ξ*_CS_ and its relatively good conductivity.^[^ ^26^ ^]^ The systematic studies about conductivity and magnetic dichroism suggest that the large Rashba‐type spin splitting originates from the bulk spin‐momentum locking by asymmetric orbital hybridization given by the ultra‐thin W layer.

## 2 Results and Discussion

### 2.1 Spin‐Orbit Torque Measurement in [Pt/Co/W]‐SLs

Our main question is what if we make an artificially non‐centrosymmetric structure for spin current generation. As mentioned earlier, Figure 1b illustrates the ABC‐type artificial SL: Ta(1.5)/[Pt(1)/Co(0.6)/W(*t*)]_10_/Pt(1)/MgO(2)/Ta(3) (all units are in nm). The sequential deposition of a monolayer of atoms with different elements introduces structural asymmetry. For this study, W thickness (*t*_W_) varies from 0.2 to 1 nm while other layers are fixed. We conducted high‐angle annular dark‐field scanning transmission electron microscopy (HAADF STEM) to confirm the quality of the superlattice with *t*_W_ = 0.6 nm. As shown in Figure 1c–f, the superlattice clearly shows the ABC‐type layered structure without severe interdiffusion. In particular, the *d*‐spacing of the W layers is about 0.27 nm which is same as the *d*‐spacing of Pt (111). This indicates that the W layers pseudomorphically grown with the FCC phase in the superlattice. We also confirm that the SLs with 0.2< *t*_W_ < 1.0 exhibit perpendicular magnetic anisotropy. DMI‐effective field of the superlattice with *t*_W_ = 0.6 nm is about 50 mT, which is a typical signature in such non‐centrosymmetric systems^[^ ^25^ ^]^ (see Supporting Information S1).

**Figure 2:**
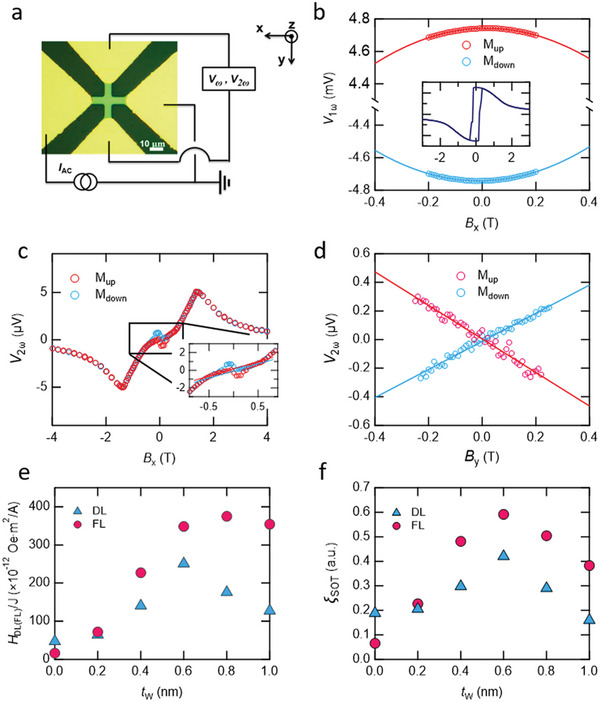
Charge‐to‐spin conversion in the [Pt/Co/W]‐SL. a) Optical image of the Hall device and measurement circuit image. b) The first harmonic (1*w*) signal with magnetization up (M_up_) and down (M_down_) for B//*x* or *y*‐axis. Inset shows the full range curve of the Hall voltage. The second harmonic Hall voltages with c) B//*x* and d) B//*y*. e) Effective fields and f) charge‐to‐spin conversion efficiency in terms of *t*_w_.

To quantify charge‐to‐spin conversion in the [Pt/Co/W]‐SL, the AC harmonic voltage measurement was performed^[^ ^27^, ^28^ ^]^ (see the schematic illustration in **Figure** **2**a). In this measurement scheme, the AC current (7 Hz) much slower than the magnetization dynamics (approximately GHz) injected into the SL wire leads to the oscillation of magnetization, thereby changing the Hall voltage if the spin current is generated in the SL, and exerting torque to the local magnetization. The first (*V*_ω_) and second harmonic Hall voltage (*V*_2ω_) are recorded by two lock‐in amplifiers at the same time. Measuring and analyzing these *V*_ω_ and *V*_2ω_ under the in‐plane magnetic field parallel to the *x* direction (*y* direction) provides quantitative values of the spin‐current‐induced DL (FL) effective field, termed *H*_DL_ (*H*_FL_).^[^ ^28^, ^29^ ^]^ Figure 2b shows *V*_ω_ of the [Pt/Co/W(0.6)]‐SL when the current density (*J*) of 7 × 10^10^A m^−2^ is applied under magnetic field swept in the *x* direction. Note that the current density flowing through the spin Hall materials (W and Pt) is estimated based on the resistivity of each material (Supporting Information S2). In the case of *V*_2ω_, severe contributions from a mixture of both thermoelectric effects and SOT are observed.^[^ ^30^ ^]^ This indicates that the pure contribution of SOT to *V*_2ω_ should be carefully subtracted for such new material systems. Measuring a full range of *V*_2ω_ curves to obtain the purely SOT‐originated signal, the thermoelectric effects arising from vertical and lateral temperature gradient were carefully subtracted as shown in Supporting Information S3. The *V*_2ω_ curves with the external magnetic field swept in the *x* and *y* directions after subtracting the other spurious contributions are shown in Figure 2c,d, respectively. *H*_DL_ and *H*_FL_ can be obtained according to the following set of equations

(1) $$H_{\mathrm{DL}}=-2\frac{\left(B_{X}\pm 2\xi B_{Y}\right)}{1-4\xi^{2}}\;\mathrm{and}\;H_{\mathrm{FL}}=-2\frac{\left(B_{Y}\pm 2\xi B_{X}\right)}{1-4\xi^{2}}$$

where

(2) $$B_{X}\equiv {\left.\left(\frac{\partial V_{2\omega }}{\partial H}/\frac{\partial^{2}V_{\omega }}{\partial H^{2}}\right)\right|}_{H∥x}\;\mathrm{and}\;B_{Y}\equiv {\left.\left(\frac{\partial V_{2\omega }}{\partial H}/\frac{\partial^{2}V_{\omega }}{\partial H^{2}}\right)\right|}_{H∥y}$$

**Figure 3:**
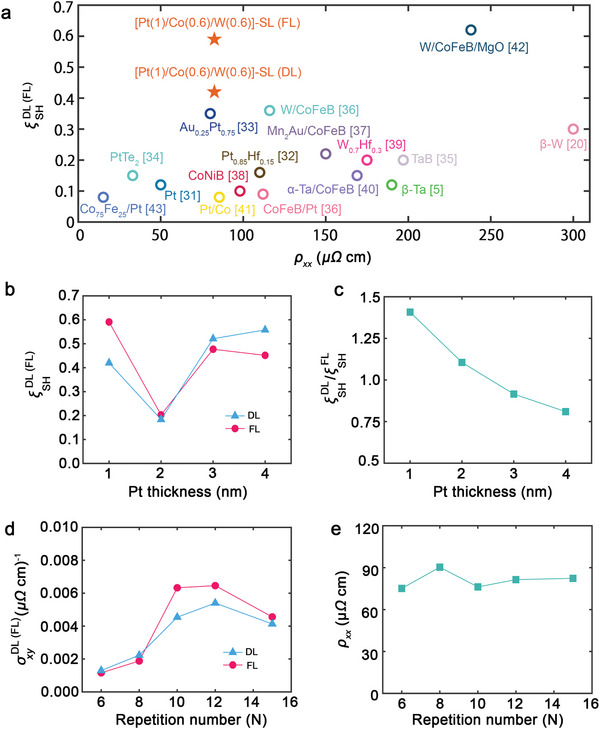
Spin Hall conductivity of the [Pt/Co/W]‐SL. a) Reported $\xi_{\mathrm{CS}}^{\mathrm{DL}(\mathrm{FL})}$ values as function of resistivity *ρ*_*xx*_ with various film stacks. b) Effective field in terms of *t*_Pt_. c) Ratio between $\xi_{\mathrm{CS}}^{\mathrm{DL}}$ and $\xi_{\mathrm{CS}}^{\mathrm{FL}}$, and d) $\sigma_{\mathrm{SH}}^{\mathrm{DL}(\mathrm{FL})}$and e) *ρ*_*xx*_ values of the [Pt/Co/W]‐SL as a function of N.

Here, *ξ* is the ratio between the planar Hall effect resistance and anomalous Hall resistance (see Supporting Information S4).^[^ ^28^ ^]^ As shown in Figure 2e, the *H*_DL_ normalized by charge current density (*H*_DL_/*J*) increases with W thickness and has the maximum when *t*_W_=0.6 nm or W(0.6), while *H*_FL_/*J* increases and becomes saturated. To investigate the charge‐to‐spin conversion ratio, the charge‐to‐spin conversion ratio ($\xi_{\mathrm{CS}}^{\mathrm{DL}(\mathrm{FL})}$) for *H*_DL(FL)_ was calculated using the relation $H_{\mathrm{DL}(\mathrm{FL})}/J\;=\;\left(\hbar /2e\right)\cdot \xi_{\mathrm{CS}}^{\mathrm{DL}(\mathrm{FL})}/M_{s}t_{\mathrm{FM}}$, where ℏ is the reduced Planck constant and *e* is the elementary charge. *M*_s_ is the saturation magnetization, and *t_FM_* is the total thickness of the ferromagnetic layer (see Figure 2f). The obtained $\xi_{\mathrm{CS}}^{\mathrm{DL}}$ of the non‐centrosymmetric [Pt/Co/W]‐SL is about twice larger than $\xi_{\mathrm{CS}}^{\mathrm{DL}}$ of the centrosymmetric [Pt/Co]‐SL. Notably, the $\xi_{\mathrm{CS}}^{\mathrm{FL}}$ of the [Pt/Co/W]‐SL reaches 0.6 which is about 10 times larger than that of [Pt/Co]‐SL. This value is also larger than other reported values for metal‐based systems,^[^ ^5^, ^20^, ^31^, ^32^, ^33^, ^34^, ^35^, ^36^, ^37^, ^38^, ^39^, ^40^, ^41^, ^42^, ^43^ ^]^ as shown in **Figure** **3**a.

In general, two cases are considered as an origin of the charge‐to‐spin conversion: bulk spin Hall and Rashba–Edelstein effects. In the framework of the bulk spin Hall effect (SHE), *ξ*_CS_ should be enhanced when *t*_W_ is thinner than the spin diffusion length ≈4.0 nm. Therefore, the 1 nm thick W single layer is not enough to provide the large $\xi_{\mathrm{CS}}^{\mathrm{DL}(\mathrm{FL})}$.^[^ ^42^ ^]^ Nguyen et al. reported that both *H*_DL_/*J* and *H*_FL_/*J* increase, reaching a maximum at 3–4 nm, which gradually decrease when Pt thickness (*t*_Pt_) is larger than 4 nm.^[^ ^31^ ^]^ If the observed enhancement of *H*_DL_/*J* and *H*_FL_/*J* in the [Pt/Co/W]‐SL is due to the bulk SHE of Pt, both values are expected to increase with a thicker *t*_Pt_ of up to ≈4 nm. Accordingly, an additional experiment was carried out to assess the dependence of the SOT on *t*_Pt_ in the [Pt(t)/Co/W(0.6)]‐SL. Figure 3b,c shows *H*_DL_/*J* and *H*_FL_/*J* with respect to *t*_Pt_. In contrast to previous studies,^[^ ^31^, ^43^, ^44^, ^45^ ^]^ both *H*_DL_/*J* and *H*_FL_/*J* reduced at *t*_Pt_ = 2 nm and increased for 2 ≤ *t*_Pt_ ≤ 4 nm. Because the increase in *H*_DL(FL)_ is related to bulk SHE when *t*_Pt_ ≥ 2 nm (see Figure 3b), the origin of the large charge‐to‐spin conversion under *t*_Pt_ = 1 nm should be distinct from the general SHE in Pt layers. Because the Rashba‐type SOC is the dominant origin of the field‐like SOT due to spin polarization at the interface,^[^ ^4^, ^46^ ^]^ the rapid enhancement of $\xi_{\mathrm{CS}}^{\mathrm{FL}}$ up to *t*_W_ = 0.6 nm in Figure 2e,f and the magnitude reversal between $\xi_{\mathrm{CS}}^{\mathrm{DL}}$ and $\xi_{\mathrm{CS}}^{\mathrm{FL}}$ around *t*_Pt_ > 2 nm shown in Figure 3b may imply that the Rashba‐type SOC is the main origin for the charge‐to‐spin conversion in the non‐centrosymmetric SLs as reported by X. Fan, et al.^[^ ^47^ ^]^ Thus, our study clearly demonstrates that the Rashba‐type origin, rather than the bulk spin‐Hall, plays an important role in charge‐to‐spin conversion.^[^ ^47^ ^]^ We also note that the upper bound of the $\xi_{\mathrm{CS}}^{\mathrm{DL}}$ can be about 0.45 for *H*_DL_/*J* in a Pt/ferromagnet/W system when we consider the previous report.^[^ ^48^ ^]^ The value should be related to the bulk origin. In general, $\xi_{\mathrm{CS}}^{\mathrm{DL}(\mathrm{FL})}$ values with Pt or W layers thinner than 1 nm should have much smaller values with Pt and W with few nanometers.^[^ ^28^, ^31^, ^47^, ^49^, ^50^, ^51^ ^]^ Since spin Hall effect and Rashba effect are companion mechanisms and usually coexist in a system, our experimental results implies that the bulk‐Rashba effect dominates the observed SOT phenomena in the [Pt/Co/W]‐SL.

As mentioned in the introduction section, bulk spin‐momentum locking is also candidate origin of such large charge‐to‐spin conversion in the non‐centrosymmetric structure. Therefore, there can be two scenarios to explain our observation, again: i) enhancement of SOT due to just simply add‐up of SOT from each interface, Pt/Co and Co/W and ii) spin‐momentum locking driven by Rashba‐type band structure in the [Pt/Co/W]‐SL. To distinguish above two possibilities, we measured SOT varying the repetition number of a single unit [Pt/Co/W] in the SL. The interfacial quality was confirmed to be independent with the repetition number (*N*) from the X‐ray reflectivity study (see Supporting Information S5). However, the estimated spin Hall conductivity $\sigma_{\mathrm{SH}}^{\mathrm{FL}}$ of the SL shows clear *N* dependence, increases up to *N* = 10, then becomes saturated, while the resistivity is *N*‐independent as shown in Figure 3d,e. The *N* dependence of $\sigma_{\mathrm{SH}}^{\mathrm{FL}}$ arises not from the simple addition of interface effect, but from construction of the band structure with increase in *N*. We also note that the saturation of $\sigma_{\mathrm{SH}}^{\mathrm{FL}}$ occurs when *N* = 10 with the total thickness of the SL around 23 nm, which is much larger than the spin diffusion length of Co, W, and Pt.^[^ ^31^, ^43^, ^44^, ^45^ ^]^ Similar trend has been reported in Tb/Co multilayer system, which shows bulk torque behavior.^[^ ^52^ ^]^ Therefore, the spin current generation is due to the spin‐momentum locking associated with the non‐centrosymmetry as found in cases of the BiTeX (X = Cl, Br, and I) systems,^[^ ^22^, ^23^ ^]^ NiMnSb^[^ ^32^ ^]^ and (Ga,Mn)As.^[^ ^33^ ^]^ In addition to the large charge‐to‐spin conversion, low conductivity is also a considerable issue in this study. Though W or Ta have relatively large $\xi_{\mathrm{CS}}^{\mathrm{DL}}$ and $\xi_{\mathrm{CS}}^{\mathrm{FL}}$ than other metals, the resistivities are high. Therefore, they are not applicable for energy‐efficient devices without material engineering.^[^ ^31^, ^53^, ^54^ ^]^ However, the observation of the large SOT with the low resistivity in the [Pt/Co/W]‐SL indicates that such an artificial SL can give benefit for developing device applications.

### 2.2 Band Structure of the SLs

**Figure 4:**
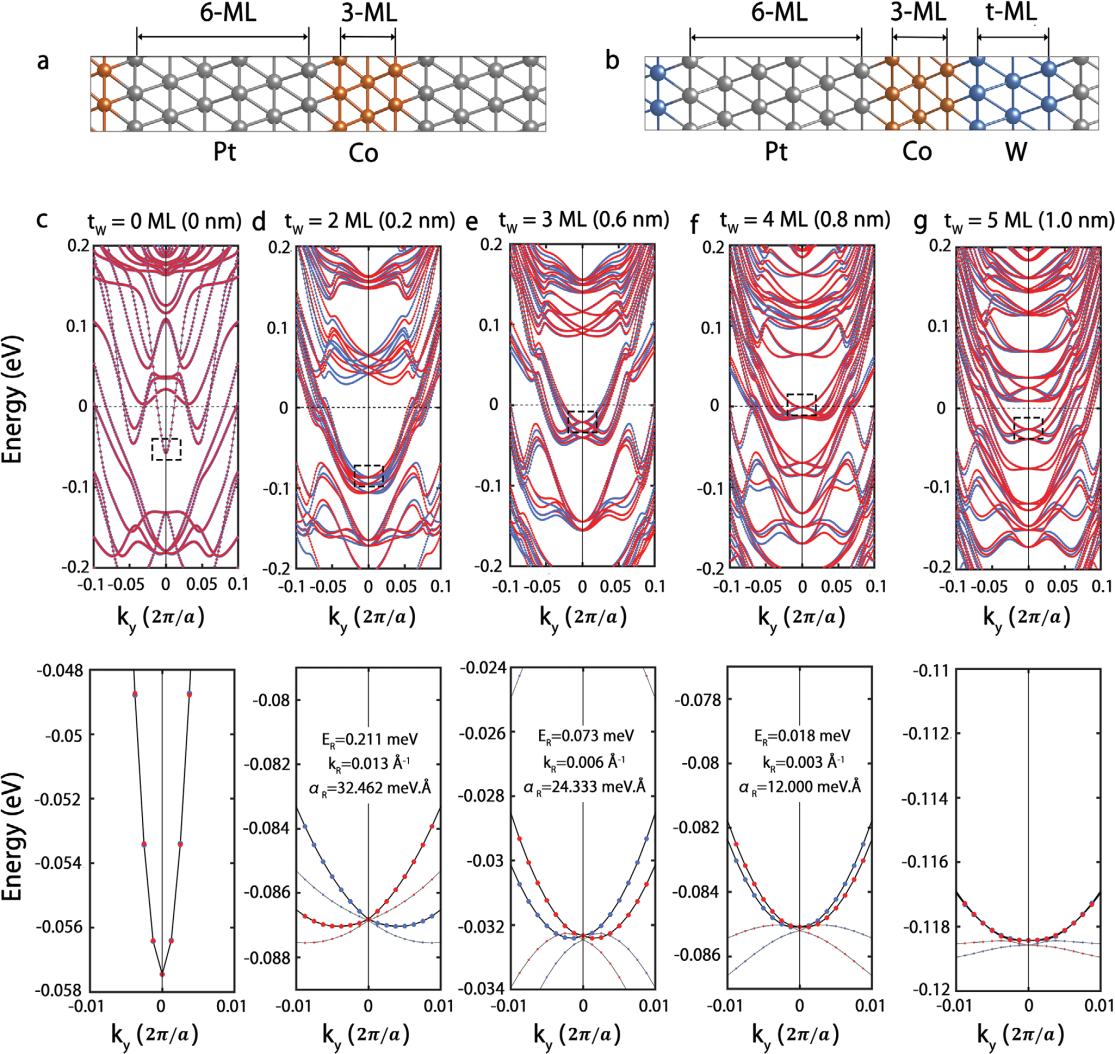
Band structure of the SLs. a,b) Schematic of unit cells used to calculate the electronic structure for the Pt/Co(111) and Pt/Co/W(111) SLs. c–g) Band structure for different in‐plane magnetization directions along +*x* and −*x* axes, plotted using respectively red and blue lines in the upper part. The lower part shows the zoom of band structure as indicated by the dashed rectangles.

**Figure 5:**
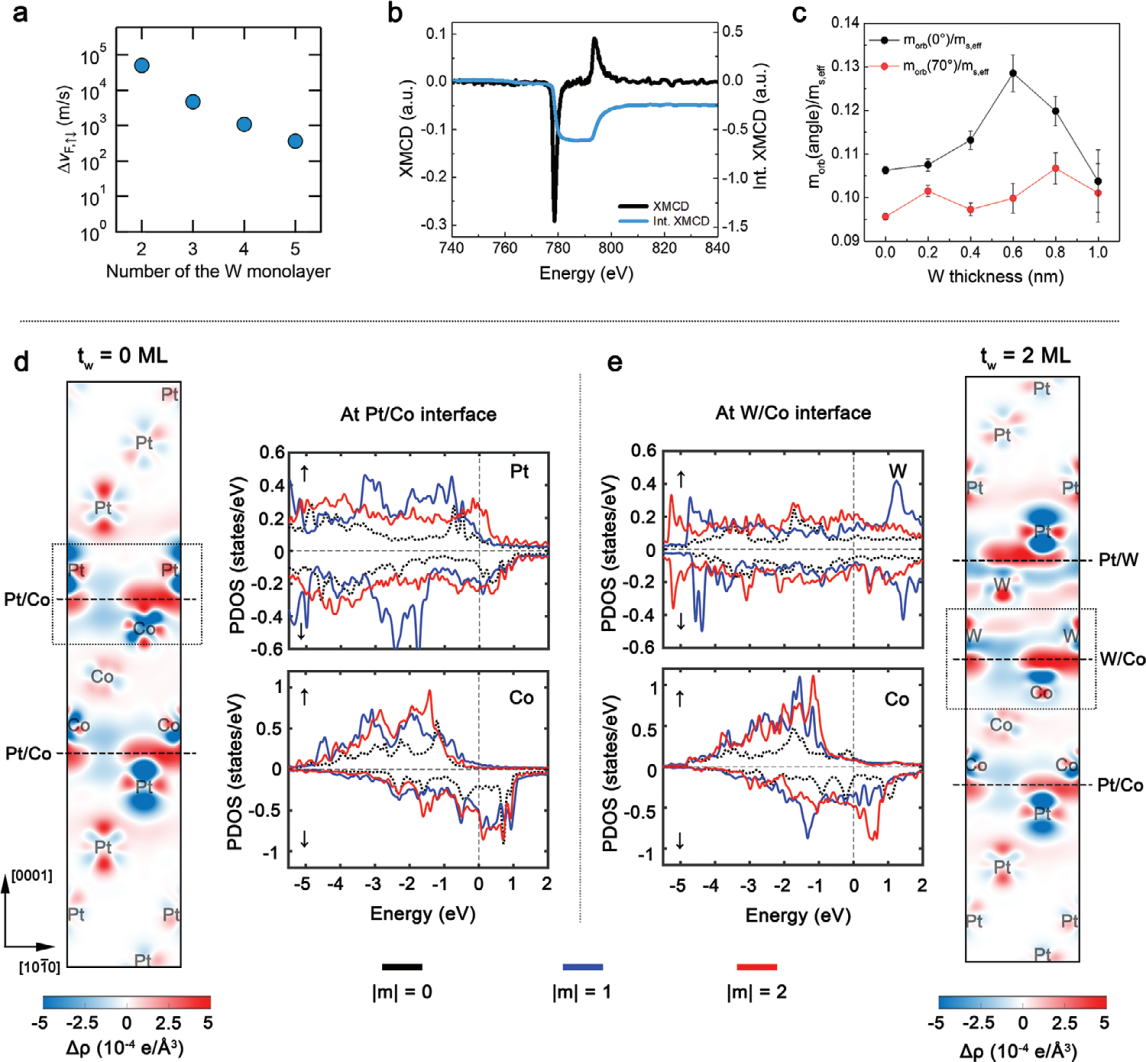
Contribution of the W orbital to the Rashba‐type splitting. a) Evaluated Δ*v*_F,↑↓_ values in terms of *t*_W_. b) XMCD and integrated XMCD spectra of the [Pt/Co (0.6)/W(0.6)]‐SL. c) Orbital‐to‐spin moment ratio in terms of *t*_W_. Differential charge density Δ*ρ* and partial density of states (PDOS) of d) [Pt/Co]‐ and e) [Pt/Co/W(2ML)]‐SLs. For charge density, the red (blue) distribution represents the charge accumulation (depletion). For PDOS, the *d* orbital states with magnetic quantum number |*m*| = 0, 1, 2 are shown in black, blue, and red, respectively.

To investigate the Rashba effect with W insertion, we show the band structures of Pt/Co(111) and Pt/Co/W(111) SL along *k*_y_ in **Figure** **4**c–g. Band structures are shown where upper panel is for wider range and lower panel is zoomed view of the dashed rectangular region. The red and blue colors indicate the corresponding bands for the in‐plane magnetization oriented along the +*x* and ‐*x* directions, respectively. The ±*x* directions are chosen to present the spin‐momentum locking as a consequence of structural asymmetry along the *z*‐axis. Two important aspects should be noted: 1) Band shift depending on the spin direction is observed owing to Rashba‐type splitting with the 2–4 ML thick W insertion. 2) The Rashba‐type splitting appears only when *t*_W_ < 4 ML. These manifest that the artificially broken symmetry induces the spin‐momentum locking observed in the experiment. The splitting is largest for *t*_W_ = 2 ML, which gradually gets smaller with *t*_W_. The *α*_*R*_ is estimated to be 32.46, 24.33, and 12.00 meV Å for *t*_W_ = 2, 3, and 4 ML, respectively. Here, 2, 3, 4, and 5 ML of W correspond to 2.47, 4.94, 7.40, and 9.84 Å thick, respectively. We also note the difference in the Fermi velocity (*v*_F_) between up and down spins near Fermi energy (*E*_F_); Δ*v*_F_ = *v*_F,↑_ − *v*_F,↓_, which governs the spin transport. Δ*v*_F_ is estimated from the simple relation *dE*/*dk* = *hv*_F_/2*π*, as plotted in **Figure** **5**a. We found that two monolayers of W leads to Δ*v*_F_ ≈ 10^5^ m/s, which decreases by an order when the W monolayer is added. This estimate is slightly different from the experiment, where *t*_W_=6Å or W (0.6) has the largest SOT. This is attributed to the situation that 1–3 atomic monolayers cannot form the full‐blanket layer using the UHV sputtering method. Nevertheless, the theoretical estimates indicate the role of W‐insertion for the Rashba‐type spin splitting.

Further analysis is provided with the orbital‐resolved band structure of [Pt/Co]‐ and [Pt/Co/W]‐SLs (see Supporting Information S6). We found that, without W, bands are dominated by the |*m*| = 1 (*d*_*yz*, *zx*_) orbital. However, with W insertion, in the [Pt/Co/W]‐SLs, |*m*| = 2 ($d_{xy,\;x^{2}-y^{2}})$ orbital becomes dominant, implying orbitals with in‐plane characters play more role in the Rashba‐type splitting.

Figure 5b,c shows the experimentally measured spin‐to‐orbital moments ratio (*m*_orb_/*m*_s,eff_) with respect to *t*_W_ using the X‐ray magnetic circular dichroism (XMCD) study (see details in Supporting Information S7). The angles 0° and 70° between X‐ray incidence and surface normal, represent out‐of‐plane ($m_{\mathrm{orb}}^{⊥}$) and in‐plane ($m_{\mathrm{orb}}^{∥}$) orbital moments, respectively. As shown in Figure 5c, the orbital moments are anisotropic; *m*_orb_(0°)/*m*_s,eff_, corresponding to $m_{\mathrm{orb}}^{⊥}$, shows similar trend, while the *m*_orb_(0°)/*m*_s,eff_, thereby $m_{o}^{∥}$, is *t*_W_‐independent. Notably, it is well‐known that the in‐plane *d* orbital |*m*| = 2 contributes to $m_{\mathrm{orb}}^{⊥}$.^[^ ^55^ ^]^ Therefore, our theoretical and experimental results demonstrate that the in‐plane *d* orbitals given by W play a major role for the Rashba‐type spin splitting.

To gain more insight, the differential charge density Δ*ρ* and partial density of states (PDOS) of [Pt/Co]‐ and [Pt/Co/W(2ML)]‐SLs are shown in Figure 5d,e, respectively. The red (blue) distribution in charge density represents the charge accumulation (depletion). One can see that the number of differential charge and the area where charge density redistributes differ significantly at the interfaces. The accumulation of charge indicates the orbital hybridization at Pt/Co, W/Co, and Pt/W layered structures. To figure out the emergence of |*m*| = 2 orbital by W‐insertion, we show PDOS of Pt, Co, and W layers at Pt/Co and W/Co interfaces for *t_W_* = 0 ML and 2 ML cases. The *d* orbitals with magnetic quantum numbers |*m*| = 0, 1, 2 are denoted in black, blue, and red, respectively. Note that from Figure 5d,e, without W, Co has large PDOS peaks of unoccupied |*m*| = 1 and |*m*| = 2 minority states at the Fermi level. However, with W insertion, the peaks of unoccupied |*m*| = 1 states shift toward the occupied states, while the unoccupied |*m*| = 2 states remain at the Fermi level. Meanwhile, W has a broad PDOS distribution for both occupied and unoccupied states. It implies that the electrons with |*m*| = 2 states of W can occupy the unoccupied |*m*| = 2 states of Co, resulting in the emergence of |*m*| = 2 orbital in [Pt/Co/W]‐SLs.

## 3 Conclusion

A combined experimental and theoretical study demonstrate that bulk Rashba‐type spin splitting is pronounced in the [Pt/Co/W]‐SL, as a result of structural asymmetry inherited from the artificial SL. $\xi_{\mathrm{CS}}$ = 0.6 from the field‐like SOT is an order larger than other metallic systems. Rashba‐type splitting is well‐manifested as dominated by in‐plane *d* orbitals both from theory and XMCD (experiment). The augmentation of the SOT with high spin Hall conductivity [7.0 × 10^5^±500 (ℏ/2e) (Ω m)^−1^] of the artificially non‐centrosymmetric system offers great potential for energy‐efficient magnetic memory devices.

## 4 Experimental Section

### Film Deposition and Device Fabrication

The [Pt/Co/W]‐SL of Ta(1.5)/[Pt(1)/Co(0.6)/W(t)]*_N_*_= 10_/Pt(1) /MgO(2)/Ta(3) (all units are in nm) was deposited on SiO_2_ substrate by magnetron sputtering. All films showed perpendicular magnetic anisotropy, confirmed by magnetic properties measurement system. To perform the DC and AC electrical measurement, the films were patterned into 5 µm × 25 µm Hall bar structure using photolithography and Ar ion milling. Then, Ti(5 nm)/Au(100 nm) electrodes were fabricated by DC magnetron sputtering and the lift‐off process.

### Structural Analysis of the Superlattice

Specimens for HAADF STEM were prepared using a focused ion beam system (Hitachi/NB 5000). The HAADF STEM images and EDS elemental mapping were performed by an FEI Themis Z microscope at Daegu Gyeongbuk Institute of Science and Technology (DGIST). The TEM is equipped with a probe spherical aberration (Cs) corrector and operated at 300 kV.

### Harmonic Measurement to Quantify SOT‐induced Effective Fields

The second harmonic response was the signal which had doubled frequency of the injected ac current (𝐼 = 𝐼_0_sin𝜔t). In the case of samples possessing perpendicular magnetic anisotropy (PMA) energy, the current induced effective field changed the anomalous Hall voltage (𝑉 = 𝐼𝑅 = (𝐼_0_sin𝜔𝑡 × 𝑅_0_sin𝜔𝑡 = 𝐼_0_𝑅_0_sin^2^𝜔𝑡 ∝ 𝐼_0_𝑅_0_cos2𝜔𝑡). Therefore, the anomalous Hall voltage contained the current induced effective fields. The ac current of 7–337 Hz which was slower enough than the magnetization dynamics (approximately GHz range) was injected to the device Kiethley 6221. The Hall signals of the first harmonic voltage (𝑉_*ω*_) and the second harmonic voltage (𝑉_2𝜔_) were obtained by the two lock‐in amplifiers (LI5630, NF corporation) at the same time.

### X‐Ray Magnetic Circular Dichroism (XMCD) Measurement

The X‐ray absorption spectra were obtained by sweeping the X‐ray photon energy in the range of Co L_3_ and L_2_ edge (740–840 keV) at 0° and 70° under a magnetic field of ± 1.9 T. The subtraction between the right‐ and left‐handed circularly polarized light corresponded to the XMCD spectra. All the measurements were carried out at BL25XU of SPring‐8.

### Computational Methods

Density functional calculations were performed using the Vienna ab initio simulation package (VASP)^[^ ^56^, ^57^ ^]^ with projector augmented wave (PAW) basis.^[^ ^58^ ^]^ The spin‐polarized generalized gradient approximation (GGA) was employed for exchange‐correlation energy with parameterization by Perdew, Becke, and Ernzerhof.^[^ ^59^ ^]^ For wave function expansion, a cutoff energy of 400 eV was chosen. For Brillouin zone summation, Γ‐centered *k* mesh of 54 × 54 × 3 was used in the Monkhort‐Pack scheme with Gaussian broadening of 0.05 eV. Spin‐orbit coupling (SOC) interaction was treated perturbatively.^[^ ^60^ ^]^ The in‐plane magnetization direction was conveniently selected along the *x*‐direction. Unit cells for [Pt/Co] and [Pt/Co/W] were constructed in hexagonal supercell of fcc (111), consisting of 6 monolayers (ML) of Pt (≈10 Å), 3 ML of Co (≈6 Å), and *t*_W_ ML of W (*t* = 2–5 ML, about 2.47, 4.94, 7.40, and 9.84 Å thick), as shown in Figure 4a,b. Calculated thicknesses of Pt, Co, and W were quite close to the experiments. The lattice constants and internal atomic coordinates were fully optimized with force criterion of 0.01 eV Å^−1^. Differential charge density at the Pt/Co and W/Co interface were calculated by Δ*ρ* = *ρ*_total_ − (*ρ*_Pt_ + *ρ*_Co_ + *ρ*_W_), where *ρ*_total_, *ρ*_Pt_, *ρ*_Co_, and *ρ*_W_ are total charge densities of the [Pt/Co]‐ and [Pt/Co/W]‐SLs, isolated Pt, Co, and W layers, respectively.

## Conflict of Interest

The authors declare no conflict of interest.

## Author Contributions

W.S.H. and T.H.H. contributed equally to this work. W.S.H., S.K, and T.O. designed the experiment. W.S.H. mainly performed the experiment and analyzed the data. T.H.H., S.H.R., and S.C.H. performed first‐principles calculations. N.‐J.L., S.L., and B.‐G.P. support the measurement and analysis. Y.S., S.K., T.L., T.I., F.A., T.I., Y.K., and T.N. helped the X‐ray magnetic circular dichroism measurement. D.K. and Y.S. helped the X‐ray diffraction studies and discussed about the results. T.M., W.S.H., S.K., and T.O. discussed the results and implications. T.O. and S.K. supervised the project.

## Supporting information

Supporting Information

## Data Availability

The data that support the findings of this study are available from the corresponding author upon reasonable request.
