# Supplementary material for: Bulk Rashba‐Type Spin Splitting in Non‐Centrosymmetric Artificial Superlattices
Source: Adv Sci (Weinh). 2023 Feb 19;10(12):2206800. doi: 10.1002/advs.202206800 (PMC10131871; doi:10.1002/advs.202206800)
Supplement: Supplementary file 1 — Supporting Information [file ADVS-10-2206800-s001.pdf]

ADVANCED SCIENCE
Open Access

Supporting Information

for *Adv. Sci.*, DOI 10.1002/advs.202206800

Bulk Rashba-Type Spin Splitting in Non-Centrosymmetric Artificial Superlattices

Woo Seung Ham, Thi Huynh Ho, Yoichi Shiota, Tatsuya Iino, Fuyuki Ando, Tetsuya Ikebuchi, Yoshinori Kotani, Tetsuya Nakamura, Daisuke Kan, Yuichi Shimakawa, Takahiro Moriyma, Eunji Im, Nyun-Jong Lee, Kyoung-Whan Kim, Soon Cheol Hong, Sonny H. Rhim, Teruo Ono and Sanghoon Kim**

Supporting Information

Bulk Rashba-type Spin Splitting in Non-Centrosymmetric Artificial Superlattices

Woo Seung Ham, Thi Huynh Ho, Yoichi Shiota, Tatsuya Iino, Fuyuki Ando, Tetsuya Ikebuchi, Yoshinori Kotani, Tetsuya Nakamura, Daisuke Kan, Yuichi Shimakawa, Takahiro Moriyma, Eunji Im, Nyun-Jong Lee, Kyoung-Whan Kim, Soon Cheol Hong, Sonny H. Rhim, Teruo Ono, and Sanghoon Kim**

W. S. Ham, Y. Shiota, T. Iino, F. Ando, T. Ikebuchi, D. Kan, Y. Shimakawa, T. Moriyma, T. Ono

Institute for Chemical Research, Kyoto University, Uji, Kyoto 611-0011, Japan

E-mail: ono@scl.kyoto-u.ac.jp

T. H. Ho, E. Im, N. J. Lee, S. C. Hong, S. H. Rhim, S. Kim

Department of Physics, University of Ulsan, Ulsan, 44610, Korea

E-mail: sanghoon.kim@ulsan.ac.kr

Y. Kotani, T. Nakamura

Japan Synchrotron Radiation Research Institute (JASRI), Sayo, Hyogo 679-5198, Japan

T. Nakamura

International Center for Synchrotron Radiation Innovation Smart, Tohoku University, Sendai 980-8572, Japan

K. W. Kim

Center for Spintronics, Korea Institute of Science and Technology (KIST), Seoul 02792, Korea

Table of Contents

S1. Magnetic anisotropies of the SLs estimated by the generalized Sucksmith-Thompson method and Dzyaloshinskii-Moriya interaction (DMI) of the [Pt/Co/W 0.6 nm]-SL

S2. Estimation of current shunting in [Pt/Co/W] superlattice

S3. Subtraction of thermoelectric effect contributions

S4. Subtraction of planar Hall contribution for quantifying SOT and confirmation of the SOT with the current-induced magnetization switching

S5. X-ray reflectivity of the [Pt/Co/W]-SLs

S6. Band structures of [Pt/Co]- and [Pt/Co/W(2ML)]-SLs with *d* orbital states

S7. Ratio between orbital to effective spin moments by XAS and XMCD spectra

S1. Magnetic anisotropies of the SLs estimated by the generalized Sucksmith-Thompson method and Dzyaloshinskii-Moriya interaction (DMI) of the [Pt/Co/W 0.6 nm]-SL

The magnetic anisotropies of SLs in this sutdy are obtained suing the generalized Sucksmith-Thompson (GST) method [S1]. In this measurement, as presented in Figure S1(a), the anomalous Hall resistance is measured with applied magnetic field in different polar angles (Here, θ = 60 ° and 80°). The Rxy vs H plots of the SL samples show typical characteristics with perpendicualr magnetic anisotropy (PMA). Then, the first and second order of magnetic anisotropy energy can be calculated as following equations,

$$2K_1^{\mathrm{eff}} + 4K_2\left(1 - m_z^2\right) = \alpha M_s H,$$

$$\alpha = \frac{m_z \sin\theta - \sqrt{1 - m_z^2}\,\mathrm{con}\theta}{m_z\sqrt{1 - m_z^2}}.$$

Here, m_z is the normalized magnetization in *z* direction, M_S is the saturation magnetization and H is external magnetic field applied in polar angle θ. Figure S1(b) shows the $\alpha M_s H$ with normalized *z*-component of magnetization. The K_1^{eff} values were obtained from the intercept and the slope by the linear fitting of $\alpha M_s H$ vs. $1 - m_z^2$ (see the values in Fig. S1(c)). As a result, the PMA in [Pt/Co/W]-SL increases with W insertion until W(0.6) and has a maximum at this thickness.

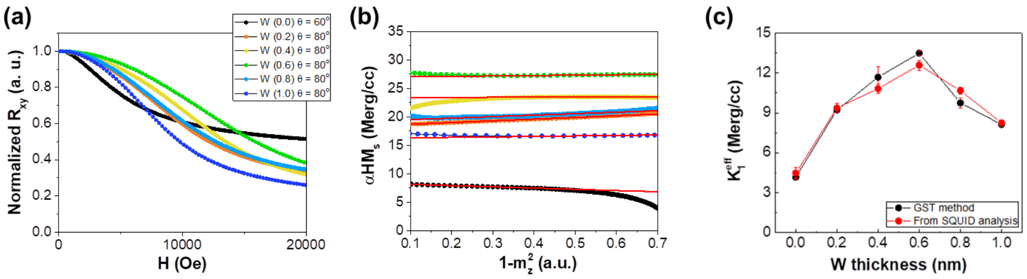

Figure S1. (a) Normalized Hall resistance (R_{xy}) with respect to the external magnetic field applied in certain angle. (b) $\alpha M_s H$ with the function of $1 - m_z^2$ in the [Pt/Co/W]-SLs. The red lines show the linear fitting. (c) The K_1^{eff} in terms of W thickness.

We could also confirm that Dzyaloshinskii-Moriya interaction (DMI) exists in the superlattice system. We measured the DMI-effective-field induced in the [Pt 1.0/Co 0.6/W 0.6 (nm)]$_{12}$ superlattice using the extended Droplet model (see ref. 25 in the main manuscript). According to the model, when

a system is perpendicularly magnetized under both B_z (//B_n) and B_x, the B_n vs. B_x curve shows threshold behavior owing to the DMI. Here, B_n is a nucleation field. From the critical field, we can estimate the DMI-induced effective field. Figure R4 shows the measured B_n in terms of B_x, and the film shows the threshold behavior marked with the yellow dotted line in the figure. Thus, the DMI-induced field B_{DMI} is about 50 mT. It has been reported that a system can have sizable DMI energy with non-centrosymmetry (see ref. 25 in the main manuscript). Therefore, DMI in our superlattice originates from the non-centrosymmetric superlattice.

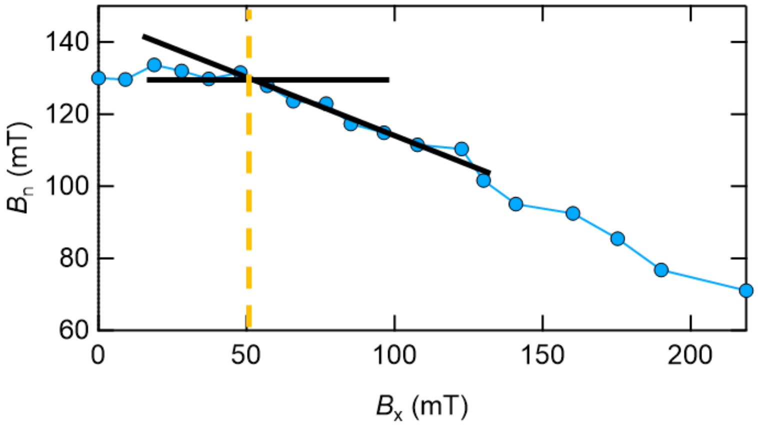

Figure S2. B_n vs. B_x curve measured with the [Pt 1.0/Co 0.6/W 0.6 (nm)]$_{12}$ superlattice. The yellow dotted line indicates the DMI-induced effective field.

S2. Estimation of current shunting in [Pt/Co/W] superlattice

The injected current density is calculated by considering the resistivity of each material. The resistivity of the materials with different thickness can be obtained from the following process. First of all, the resistivity of Ta(1.5 nm)/X (t nm)/MgO(2 nm)/Ta(3 nm) structure is measured (X = Pt (1 ~ 5 nm), Co (0.6 nm), and W (0.2 ~ 1 nm)). Then, the resistivity of Ta(1.5 nm) is measured in Ta(1.5 nm)/MgO(2 nm)/Ta(3 nm) structure and subtracted under the assumption of the parallel circuit. All the films are fabricated in the microstrip and measured the resistivity. Figures S3 (a) and (b) show the resistivity of different W and Pt thickness measured in Ta(1.5 nm)/W(t) or Pt(t)/MgO(2 nm)/Ta(3 nm), respectively. The resistivity of the materials is consistent with the previous research measured in similar thickness [S2, S3]. The fairly high resistivity of W ($\geq 100\ \mu\Omega$ cm) suggests that W could have a mixture of alpha and beta phase [S4]. On the other hand, XRD spectrum cannot clearly distinguish α and β-W in [Pt/Co/W]-SL (See Fig. S3). As mentioned in the main text, the current density (J) in quantifying $H_{DL}(H_{FL})/J$ based on the current flowing only in spin Hall materials (W and Pt). However, we would like to note that we confirmed that the trend of $H_{DL}(H_{FL})/J$ is not changed even we do not considering the current shunting effect. (*i.e.*, current flowing throughout the whole structure).

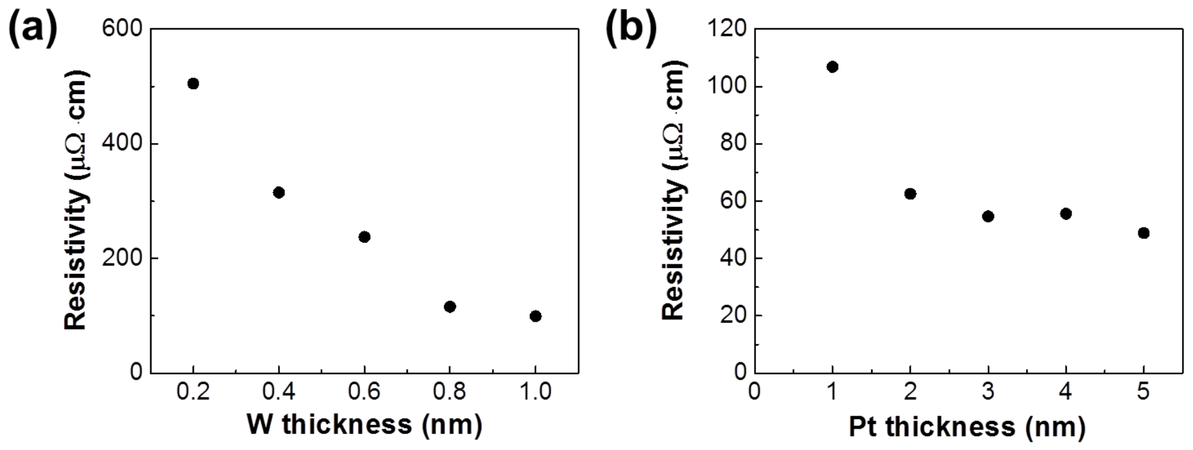

Figure S3. (a) The resistivity of W with different thickness. (b) The resistivity of Pt with the function of Pt thickness.

S3. Subtraction of thermoelectric effect contributions

In addition to the PHE, the thermoelectric effect due to the temperature gradient in *x* and *z* direction (∇T_x and ∇T_z) contribute to the 2nd harmonic voltage [S5, S6]. In particular, ∇T_z which is caused by the different thermal conductivity between the wafer and the air affects to the slope of $V_{2\omega}$ when the field is applied in *x* direction ($V_{2\omega} \sim \nabla T_z \times m_x$), thereby easily overestimating H_{DL}. Therefore, one must subtract this contribution from the raw data of $V_{2\omega}$. Actually, in the raw data, the thermoelectric effect due to anomalous Nernst effect (ANE) and spin Seebeck effect (SSE) is dominant over DL-SOT under high magnetic field (= 9 T) in this series of samples as shown in the Fig. S4 (a). (The example is only shown for the case of [Pt/Co/W(0.6)]-SL.) Therefore, the maximum value of $V_{2\omega}$ is considered entirely from thermoelectric effects because the DL-SOT becomes ‘0’ under strong magnetic field. The thermoelectric signal is reconstructed from the first harmonic data as shown in Figure S4 (b). The $V_{2\omega}$ due to pure DL-SOT is obtained as in Figure S4 (c) after subtracting the reconstructed ANE signal from the raw data. The $V_{2\omega}$ from thermoelectric effects are plotted in Figure S4 (d) with the function of W thickness at 10 mA except the case of W(0.0). (The thermoelectric effects contribution to $V_{2\omega}$ in W(0.0) is recorded at 1 mA applied to the 500 nm-width Hall bar structure.)

Especially, we would like to note that most of the previous research investigating spin-orbit torque in magnetic multilayer system overlooked these thermoelectric contributions, thereby obtaining misleading value of spin Hall angle [S7, S8]. Since both ANE and SSE are significant in multilayer system are significant in magnetic multilayer system [S9, S10], one needs to be cautious about quantifying the spin-orbit torque.

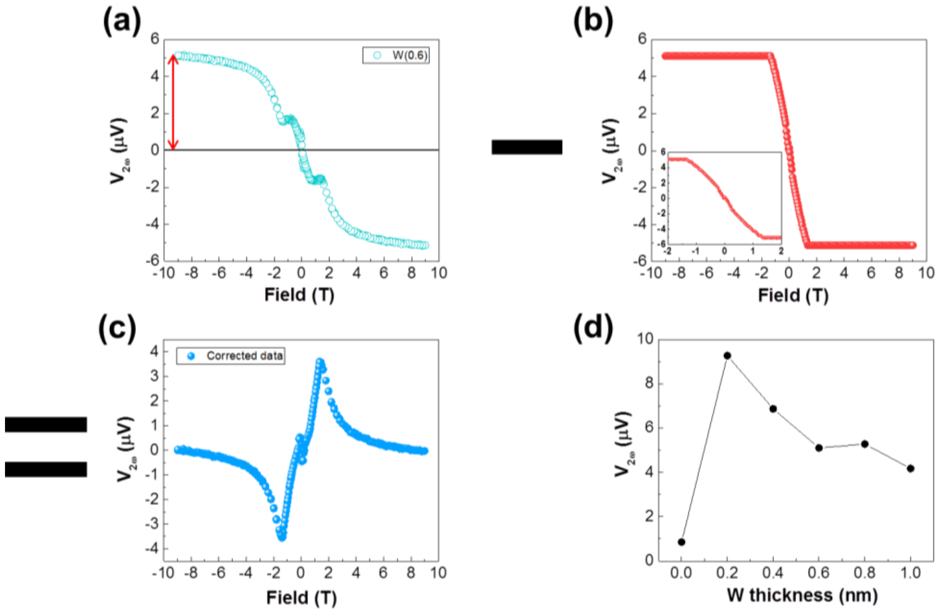

Figure S4. (a) Raw data of $V_{2\omega}$ in [Pt/Co/W(0.6)]-SL. (b) Reconstructed $V_{2\omega,\nabla T}$ from (a) and the V_{ω}. (c) Corrected data after subtraction. (d) $V_{2\omega,\nabla T}$ in the series of W thickness variation.

S4. Subtraction of planar Hall contribution for quantifying SOT and confirmation of the SOT with the current-induced magnetization switching

In order to quantify the spin-orbit effective field, one needs to consider about the contribution from the planar Hall effect (PHE). The planar Hall resistance ($\mathrm{R_{PHE}}$) is the transverse component of the anisotropic magnetoresistance (AMR). During the harmonic Hall voltage measurement, this PHE also generates transverse Hall voltage [S11]. Therefore, the PHE correction is necessary when analyzing SOT effect. Figure S5 (a) shows the angle scan of the Hall voltage (R_{xy}) measured in [Pt/Co]-SL. During the measurement, high enough magnetic field (= 5 T) than anisotropy field (H_K^{eff}) of all samples applied in *x-y* plane. The difference between maximum and minimum of R_{xy} corresponds to $2R_{\mathrm{PHE}}$. Figures S5 (b) and (c) show R_{PHE} and anomalous Hall resistance (R_{AHE}) in [Pt/Co/W(t)]-SL and [Pt(t)/Co/W(0.6)]-SL, respectively. $\mathrm{R_{PHE}}$ is plotted in negligible compared to the anomalous Hall resistance ($\mathrm{R_{AHE}}$) ($\xi = \frac{R_{\mathrm{PHE}}}{R_{\mathrm{AHE}}} < 5\%$, at best) in [Pt/Co/W(t)]-SL, except W = 0 nm (*i.e.*, [Pt/Co]-SL). However, R_{PHE} is comparable to R_{AHE} when the Pt thickness is changed in [Pt(t)/Co/W(0.6)]-SL. In all cases, the spin-orbit effective fields can be calculated by the following equations with considering the planar Hall effect contribution: $\mathrm{H_{DL}} = -2\,\frac{(\mathrm{B_X} \pm 2\xi \mathrm{B_Y})}{1-4\xi^2}$ and $\mathrm{H_{FL}} = -2\,\frac{(\mathrm{B_Y} \pm 2\xi \mathrm{B_X})}{1-4\xi^2}$, where $\mathrm{B_X} \equiv \left(\frac{\partial \mathrm{V_{2\omega}}}{\partial \mathrm{H}} / \frac{\partial^2 \mathrm{V_{\omega}}}{\partial \mathrm{H}^2}\right)\Big|_{\mathrm{H \parallel x}}$ and $\mathrm{B_Y} \equiv \left(\frac{\partial \mathrm{V_{2\omega}}}{\partial \mathrm{H}} / \frac{\partial^2 \mathrm{V_{\omega}}}{\partial \mathrm{H}^2}\right)\Big|_{\mathrm{H \parallel y}}$, respectively.

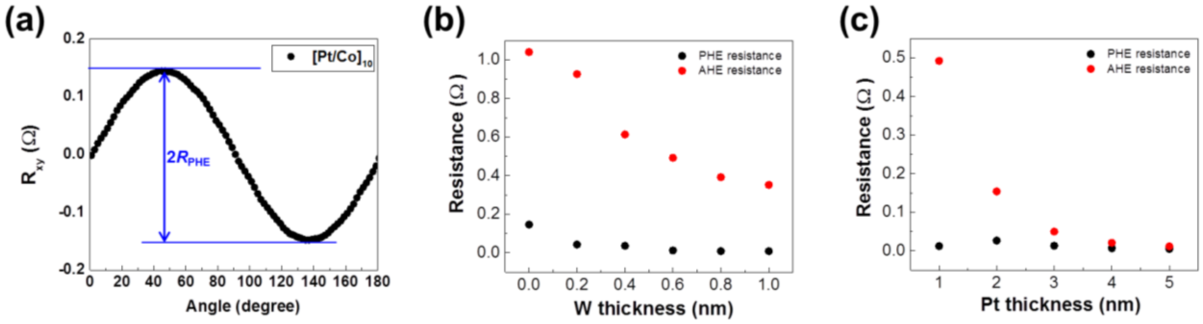

Figure S5. (a) The angle dependence of planar Hall resistance in [Pt/Co]-SL. (b) AHE and PHE resistance in [Pt/Co/W(t)]-SL with various W thickness. (c) AHE and PHE resistance in [Pt(t)/Co/W(0.6)]-SL with different Pt thickness.

We also compared the SOT effective values with and without PHE correction as listed in Table S1. We can find that the values are different from each other, but the trend is the same; $\Delta\xi_{FL}/\Delta\xi_{DL} < 1$ when $t_{Pt} > 2$ nm, otherwise $\Delta\xi_{FL}/\Delta\xi_{DL} > 1$. The obtained ξ values are similar to previously reported values as displayed in Fig. 3 of the main manuscript. In these points of view, we would like to

carefully emphasize that our main argument about the mechanism transition from Rashba to bulk spin Hall effect is reasonable.

Table S1. R_{PHE}, R_{PHE}, R_{PHE}, R_{PHE}/R_{AHE} and $\Delta\xi_{FL}/\Delta\xi_{DL}$ values in terms of t_{Pt}.

t_{Pt}	R_{PHE}/R_{AHE}	$\Delta\xi_{FL}/\Delta\xi_{DL}$ w/ PHE correction	$\Delta\xi_{FL}/\Delta\xi_{DL}$ w/o PHE correction
1 nm	0.03	1.41	1.46
2 nm	0.17	1.11	1.23
3 nm	0.26	0.92	0.76
4 nm	0.35	0.81	0.36

We additionally conducted the SOT switching experiment with the $[Pt(1.0)/Co(1.0)/W(0.6)]_{12}$ superlattice. We successfully observed the SOT-induced magnetization switching behavior of the device under B_x=±47.5 mT as shown in Fig. S6. The observed switching polarity depends on the B_x direction, which indicates the SOT-induced switching. For both cases, the switching current is ~72 mA, whose current density is $\sim 1.0 \times 10^{11}$ A/m^2. Adopting the anisotropy B_k=1.5 T, the estimated SOT-switching efficiency $[(J_c/B_k)^{-1}]$ is 1.5 ($\times 10^{-7}$ Oe $\cdot$ m^2/A) which is much larger than the Pt/Co bilayer cases ~0.1 [S12]. This trend (enhancement in the SOT-switching efficiency) is consistent with our 2nd harmonic measurement results. Therefore, the second harmonic measurement results are reliable.

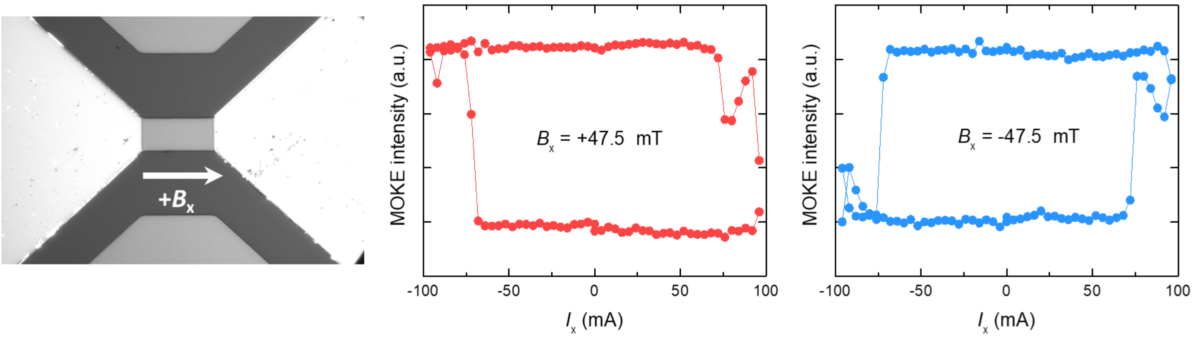

Figure S6. (left) An optical image of the device. The SOT-induced switching curve of the superlattice under (middle) B_x=47.5 mT and (right) -47.5 mT.

S5. X-ray reflectivity of the [Pt/Co/W]-SLs

The series of superlattices with accurately controlled thickness in a mono-atomic level with a sharp interface are the essential necessity of this research. Therefore, the sputtering power and time are delicately manipulated. For example, when an element having a large mass such as W is deposited on the Co, the interface between Co/W could be damaged because of the bombardment energy during the sputtering process. Therefore, the sputtering power is optimized to create the superlattice with less damaged interface which is deduced from the roughness in XRR spectra. Figure S7 shows the XRR spectra from each superlattice with varied W thickness and their fitting. From the fitting, the thickness and roughness are obtained as Table S2. Here, the numbers in the blanket next to each material show the desired thickness. The thicknesses calculated from the fitting is well matched with the targeted values. The roughnesses are around 3 ~ 4 Å range for Ta, Pt, and Co. This value is reasonable considering the atomic radii for each material (Ta = 1.45 Å, Pt = 1.35 Å, and Co = 1.35 Å) [S13], indicating 2 ~ 3 monolayers of deviation from ideally flat interface. On the other hand, the roughness of W is saturated around W(0.6 nm) which may indicate that the W forms an island in the lower thickness regime and constant roughness at higher thickness.

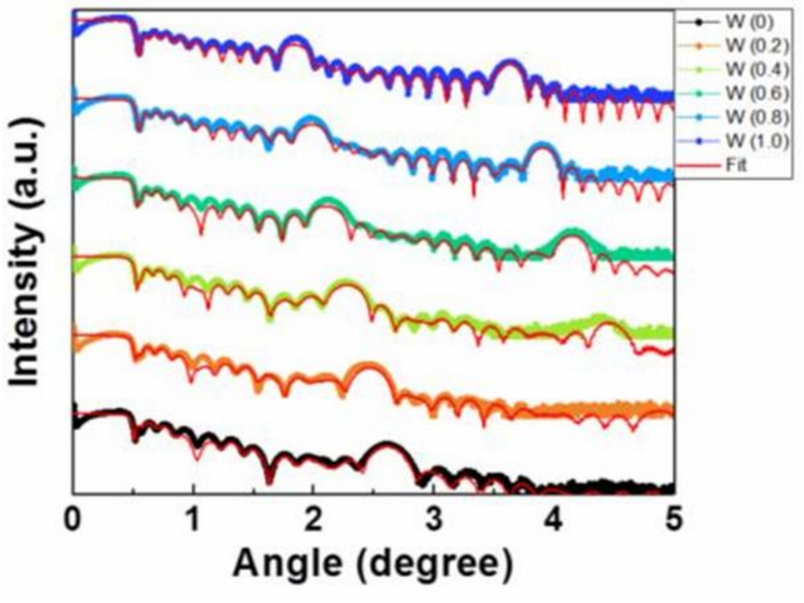

Figure S7. X-ray reflectivity (XRR) of the [Pt/Co/W(t)]-SLs.

Table S2. Thickness and roughness estimated from XRR fitting. For Ta, Pt and Co. The error is calculated from the standard deviation.

Material (nm)	Thickness (nm)	Roughness (nm)
Ta (1.5)	1.588 ± 0.077	0.448 ± 0.096
Pt (1.0)	0.970 ± 0.034	0.283 ± 0.029
Co (0.6)	0.612 ± 0.059	0.431 ± 0.086
W (0.2)	0.185	0.010
W (0.4)	0.396	0.178
W (0.6)	0.568	0.565
W (0.8)	0.770	0.521
W (1.0)	0.997	0.573

S6. Band structures of [Pt/Co]- and [Pt/Co/W(2ML)]-SLs with *d* orbital states

Figure S8 shows band structure of [Pt/Co]- and [Pt/Co/W(2ML)]-SLs with d orbital projection of d_{z^2}, $d_{yz,zx}$, and d_{xy,x^2-y^2}, whose magnetic quantum numbers are $m = 0$ (black), $|m| = 1$ (green), and $|m| = 2$ (orange), respectively. Without W, in [Pt/Co]-SL, the $|m| = 1$ orbital dominate. However, with W insertion, in the [Pt/Co/W]-SLs, $|m| = 2$ orbital becomes dominant, implying orbitals with in-plane characters play essential role in the Rashba-type splitting.

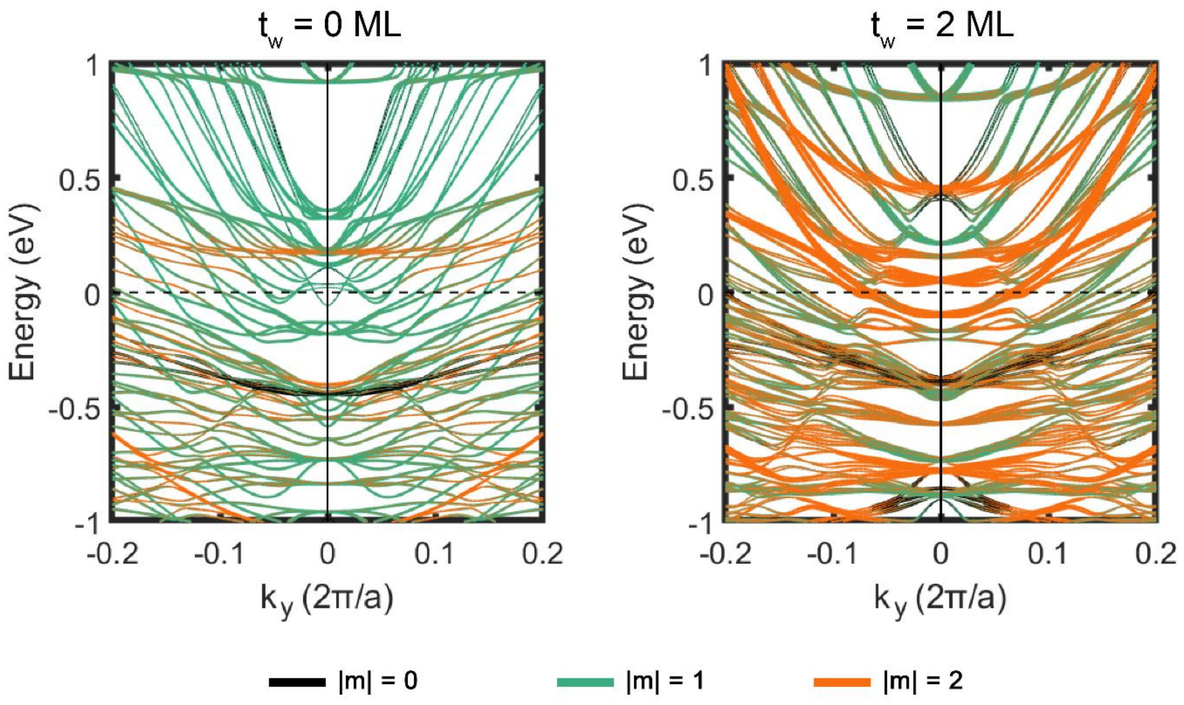

Figure S8. Band structures of [Pt/Co]- and [Pt/Co/W(2ML)]-SLs with *d* orbital states projected where magnetic quantum number $|m| = 0, 1, 2$ are shown in black, green, and orange, respectively.

S7. Ratio between orbital and effective spin moments estimated from XAS and XMCD spectra

X-ray absorption spectra (XAS) are recorded in a range between Co L_3 and L_2 edge (740 ~ 840 keV) with normal and grazing incidence at 0 º and 70º. The applied magnetic field is ± 2 T which is high enough to saturate the magnetization. The parallel and antiparallel magnetic field to the film plane provides different absorption coefficient of the photon. The difference between the right and the left circularly polarized spectra corresponds to the XMCD spectrum. All the measurements are performed in the ultrahigh vacuum and at the room temperature. The backgrounds of XAS and XMCD were subtracted using a linear function and an arctangent step function. The results after the subtraction of XAS and XMCD spectra are shown in Figure S9(a) and (b). From the integrated XMCD and XAS spectra, the orbital moment contribution to the effective spin moment $(m_{\mathrm{o}}(\theta)/m_{\mathrm{s,eff}})$ can be calculated by the following equations usually called sum rules [S14] as follows;

$$m_{orb} = -\frac{4\int_{L_3+L_2}(\mu_+ - \mu_-)d\omega}{3\int_{L_3+L_2}(\mu_+ + \mu_-)d\omega}(10 - n_{3d}) = -\frac{4q}{3r}(10 - n_{3d}),$$

$$m_{spin} = -\frac{6\int_{L_3}(\mu_+ - \mu_-)d\omega - 4\int_{L_3+L_2}(\mu_+ - \mu_-)d\omega}{\int_{L_3+L_2}(\mu_+ + \mu_-)d\omega}(10 - n_{3d})\left(1 + \frac{7\langle T_z\rangle}{2\langle S_z\rangle}\right)^{-1}$$
$$= -\frac{6p - 4q}{r}(10 - n_{3d})\left(1 + \frac{7\langle T_z\rangle}{2\langle S_z\rangle}\right)^{-1},$$

$$\frac{m_{orb}}{m_{spin}} \cong \frac{2q}{(9p - 6q)}$$

where, n_{3d} is 3d electron occupation number of Co, $\langle T_z\rangle$ is the expectation value of the dipolar operator and $\langle S_z\rangle$ is $m_{\mathrm{spin}}/2$ in Hartree atomic units. Here, the value of p and q are the integrals over L_3 edge and both L_3 and L_2 edges, respectively in XMCD spectra. The value of r can be calculated from the integration of L_3 and L_2 edges in XAS spectra. In addition, we quantified and confirmed that the $\langle T_z\rangle$ is much smaller compared to $\langle S_z\rangle$. Therefore, the orbital moment to the effective spin moment can be calculated by using last equation with neglecting the contribution from $\frac{7\langle T_z\rangle}{2\langle S_z\rangle}$.

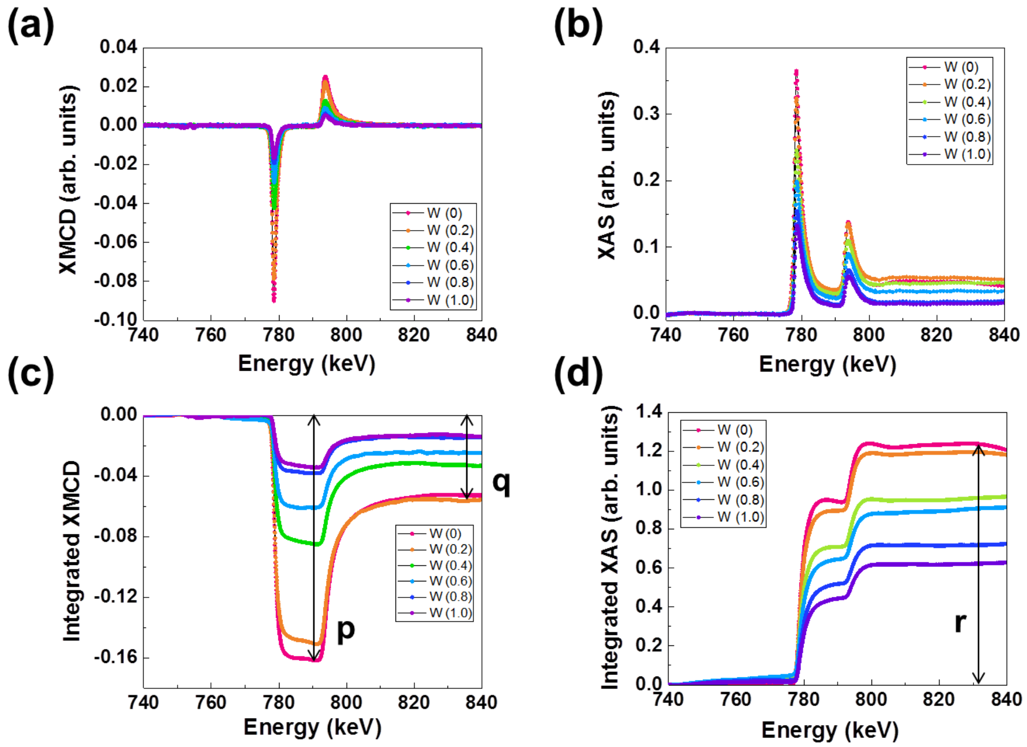

Figure S9. (a), (b) XMCD and XAS spectra in [Pt/Co/W(t)]-SL with different W thickness, respectively. (c), (d) The integrated curves of XMCD and XAS spectra in [Pt/Co/W(t)]-SL.

References

[S1] W. Sucksmith, J. E. Thompson, *Proc. R. Soc. Lond. A. Math. Phys. Sci.*, **225**, 362 (1954).

[S2] Nguyen, M.-H., Ralph, D. C. & Buhrman, R. A., *Phys. Rev. Lett.* **116**, 126601 (2016).

[S3] Lee, J. W., Oh, Y.-W., Park, S.-Y., Figueroa, I. A., Laan., van der G., Go, G., Lee, K.-J. & Park, B.-G., *Phys. Rev. B.* **96**, 064405 (2017).

[S4] Zhang, C., Fukami, S., Watanabe, K., Ohkawara, A., DuttaGupta, S., Sato, H., Matsukura, F. & Ohno, H., *Appl. Phys. Lett.* **109**, 192405 (2016).

[S5] Avci, C. O., Garello, K., Gabureac, M., Ghosh, A., Fuhrer, A., Alvarado, S. F. & Gambardella, P., *Phys. Rev. B.* **90**, 224427 (2014).

[S6] Ghosh, A., Garello, K., Avci, C. O., Gabureac, M. & Gambardella, P., *Phys. Rev. Appl.* **7**, 014004 (2017).

[S7] Huang, K.-F., Wang, D.-S., Lin, H.-H. & Lai, C.-H., *Appl. Phys. Lett*. **107**, 232407 (2015).

[S8] Jamali, Mahdi., Narayanapillai, K., Qiu, Xuepeng., Loong, L. M., Manchon, A. & Yang, H., *Phys. Rev. Lett.* **111**, 246602 (2013).

[S9] Lee, K.-D., Kim, D.-J., Lee, H.-Y., Kim, S.-H., Lee, J.-H., Lee, K.-M., Jeong, J.-R., Lee, K.-S., Song, H.-S., Sohn, J.-W., Shin, S.-C. & Park, B.-G., *Sci. Rep.* **5**, 10249 (2015).

[S10] Ramos, R., Kikkawa, T., Aguirre, M. H., Lucas, I., Anadón, A., Oyake, T., Uchida, T., Adachi, H., Shiomi, J., Algarabel, P. A., Morellón, L., Maekawa, S., Saitoh, E. & Ibarra, M. R., *Phys. Rev. B.* **92**, 220407(R) (2015).

[S11] Hayashi, M., Kim, J., Yamanouchi, M., & Ohno, H., *Phys. Rev. B.* 89, 144425 (2014).

[S12] Kang, M. G., Choi, J. G., Jeong, J., Park, J. Y., Park, H. J., Kim, T., Lee, T., Kim, K.-J., Kim, K.-W., Oh, J. H., Viet, D. D., Jeong, J.-R., Yuk, J. M. Park, J., Lee, K.-J. & Park, B. G. (2021). *Nat. Commun.* 12, 7111 (2021).

[S13] J. C. Slater, Atomic Radii in Crystals. *J. Chem. Phys.*, **41**, 3199 (1964).

[S14] Chen, C. T., Idzerda, Y. U., Lin, H.-J., Smith, N. V., Meigs, G., Chaban, E., Ho, G. H., Pellegrin, E. & Sette, F., *Phys. Rev. Lett.* **75**, 152 (1995).
